# Relationship between Knowledge and Attitude toward Oral Function in Middle-Aged and Older Adults

**DOI:** 10.1155/2022/3503644

**Published:** 2022-08-18

**Authors:** Haruka Nakata, Yuichi Masaki, Yuri Watanabe, Mai Ohkubo, Tetsuya Sugiyama, Kenichiro Kobayashi

**Affiliations:** ^1^Department of Dental Hygiene, Tokyo Dental Junior College, 2-9-18 Misakicho Tokyo, Chiyoda-Ku 101-0061, Japan; ^2^Kobayashi Dental Clinic, Tokyo, Japan; ^3^Department of Oral Health and Clinical Science, Division of Dysphagia Rehabilitation, Tokyo Dental College, 2-9-18 Misakicho Tokyo, Chiyoda-Ku 101-0061, Japan; ^4^Division of General Dentistry, Division of Dysphagia Rehabilitation, Chiba Dental Center, Tokyo Dental College, 1-2-2 Masago Mihama-Ku, Chiba 261-8502, Japan

## Abstract

Oral hypofunction is the stage at which recovery can be expected with proper diagnosis, management, and motivation before oral dysfunction occurs. The knowledge and attitude toward oral function can influence the maintenance and improvement of oral function. However, whether middle-aged and older adults with declining oral function have knowledge of their oral function and how this knowledge and their attitude affect their oral function are unclear. Therefore, we aimed to examine (1) the relationship between knowledge and attitude toward oral function and hypofunction in individuals with suspected oral hypofunction and (2) changes in knowledge and attitude toward oral function through evaluation and education. Participants aged ≥50 years were enrolled during their first community dental clinic visit. A questionnaire assessment of knowledge and attitudes before and after oral function evaluation was performed. The oral function was initially assessed with seven criteria: oral hygiene; oral dryness; occlusal force; tongue pressure; tongue-lip motor, masticatory, and swallowing function. Associations between knowledge and attitudes and their changes were statistically analyzed. Fifty-nine participants (93.7%) were unaware of “oral hypofunction.” Associations between knowledge and attitudes and their changes in the negative to positive response groups, from 86.4% and 61.0% to 6.8% and 25.4%, respectively, after oral function evaluation, indicated that participants understood their oral function and the need for training. Middle-aged and older individuals with poor knowledge and attitudes were more likely to have a worse oral function; however, their knowledge and attitudes toward oral function could be improved through oral function assessment and education.

## 1. Introduction

The global population is rapidly aging. The number of elderly individuals worldwide is projected to increase to 1.5 billion by 2050 [1], with the proportion of individuals aged ≥65 years increasing to 16.0% in 2050 [1]. People aged ≥65 years comprised 28.9% of the Japanese population in 2022, which is predicted to rise to 38.1% by 2060 [2]. The average life expectancy in 2019 was 86.9 and 81.5 years for women and men, respectively, which is among the highest in the world. The gap between average and healthy life expectancies was 11.4 and 8.9 years for women and men, respectively [3], which indicates the period of ill-health with restrictions on everyday living activities. There is a particularly large gap between a healthy life and an average life expectancy in Japan. With the aging population and the declining birth rates, the lack of caregivers and increased nursing-care costs are becoming serious problems [4].

Aging is associated with a decrease in muscle mass and a decline in nervous system function, which leads to physical changes throughout the body, including the oral cavity [5]. Changes to the oral cavity caused by aging include a decrease in the number of teeth and changes in the dentition, occlusion, and temporomandibular joints [6]. These factors may lead to a decline in masticatory function. Malocclusion and changes in temporomandibular joints can also affect mastication [7, 8]. This decline in masticatory function may be influenced by physical factors in addition to the decline in tooth numbers and muscle strength [9]; thus, a more comprehensive review is essential in the evaluation of oral function.

Enjoying a meal is a pleasure in life, particularly for older adults. Thus, adults need to be able to eat properly even at an advanced age. The mouth is also involved in conversation, expression, and sensuousness, not only food intake. In time, the physical and mental activities of older adults decrease, possibly leading to dementia [10] or a bedridden state [11]. The decline in oral function (ORF) progresses in stages along with systemic function decline. The Japanese Society of Gerodontology (JSG) divided ORF into four stages: healthy state, oral frailty, oral hypofunction, and oral dysfunction [12]. To prevent the progression of these stages, implementing a program corresponding to each stage is recommended. Oral dysfunction causes difficulty in eating, diseases, and disturbances in social life. This may influence the healthy life expectancy of older adults. Improving the ORF of older individuals can prevent these consequences and lead to a decrease in the need for long-term care.

Frailty is an important consideration in geriatric medicine. Defined as a state of increased vulnerability to internal and external stresses due to aging or disease [13, 14], frailty is characterized by reversibility or the ability to return to a healthy state with appropriate intervention. As frailty progresses, the risk of developing irreversible, serious diseases and disabilities may increase. Similarly, oral frailty and hypofunction emerge with oral dysfunction among many aspects of declining ability [12]. Initially, the symptoms may be trivial (slurred speech and spilling of food); however, as the condition worsens, it can lead to disorders such as masticatory dysfunction and dysphagia, thereby affecting overall health. Therefore, prompt and appropriate intervention is necessary.

JSG published a position paper in 2018 [12] proposing “oral hypofunction” as the stage at which recovery can be expected with proper diagnosis, management, and motivation before oral dysfunction occurs. Motivation has a great impact on people's ability to take action. Motivational interviewing can improve the periodontal status of patients with periodontal disease [15] or the oral health status of pregnant women [16]. Education regarding knowledge and attitude towards oral function (KAOF) or health may lead to improved patient behavior because there is a relationship between oral literacy and oral health status [17, 18]. This may be true for patients with periodontal disease and pregnant women, as well as middle-aged and older adults with oral hypofunction. The first step toward good KAOF is to be aware of one's ORF status and the positive impact that improving ORF has on one's overall health. This is extremely important: Having good KAOF and taking appropriate actions from the middle-age stage is the main way to prevent oral hypofunction in the future. The same is true when one is in an aging state: Good KAOF will lead to the maintenance and improvement of ORF, while those with poor KAOF are at a very high risk of functional decline due to their lack of interest in ORF. However, whether middle-aged and older adults with declining ORF have knowledge of their ORF and how this knowledge and their attitude affect their ORF are unclear. In addition, few studies reported the improvement in KAOF of dental practitioners through evaluation and education. We hypothesized that those with poor KAOF may have lower ORF and that assessment and education related to ORF would improve KAOF. Therefore, to test this hypothesis, we aimed to examine (1) the relationship between KAOF and ORF in patients with suspected oral hypofunction and (2) changes in KAOF through evaluation and education.

## 2. Materials and Methods

### 2.1. Participants

We included patients aged ≥50 years who first visited a community dental clinic between November 2019 and May 2021. After verbally explaining the study purpose to participants and providing the written text, 63 patients provided consent to participate and were included in this cohort study. Exclusion criteria included difficulty in communication and diagnosis of dysphagia. The study protocol was approved by the ethics committee of Tokyo Dental College (approval no: 948) and conformed to the provisions of the Declaration of Helsinki (as revised in Brazil 2013).

### 2.2. KAOF Questionnaire

**Figure 1:**
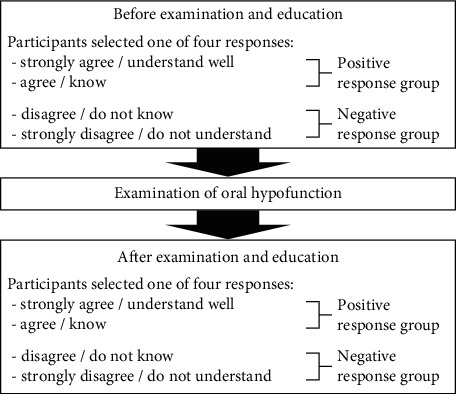
Schematic flow diagram of the study.

The KAOF was evaluated before and after evaluating the ORF, using a questionnaire on ORF (Table 1) in Japanese developed by the authors. The respondents answered each question by selecting one of four responses: “strongly agree/understand well,” “agree/know,” “disagree/do not know,” and “strongly disagree/do not understand.” The answers “strongly agree/understand well” and “agree/know” were classified as positive KAOF, while “disagree/do not know” and “strongly disagree/do not understand” were judged as negative KAOF (Figure 1). Data of participants were excluded if the questionnaire was not completed post evaluation.

### 2.3. Oral Function

ORF was assessed at the first visit based on seven criteria for diagnosing oral hypofunction: oral hygiene, oral dryness, occlusal force, tongue-lip motor function, tongue pressure, masticatory function, and swallowing function (Table 2). Oral hypofunction was diagnosed when three or more of the seven criteria were fulfilled.

#### 2.3.1. Oral Hygiene

Oral hygiene was evaluated based on tongue coating. The degree of tongue coating was assessed through visual inspection using the tongue coating index (TCI) [19].

#### 2.3.2. Oral Dryness

Oral dryness was evaluated using an oral moisture checker (Mucus, Life Co., Ltd., Saitama, Japan), which was used to measure mucosal wetness at the center of the dorsal surface of the tongue [20].

#### 2.3.3. Occlusal Force

The occlusal force of the dentition for 3 s of clenching in the intercuspal position was measured using a pressure-indicating film. For denture wearers, measurements were performed with the dentures in place [21].

#### 2.3.4. Tongue-Lip Motor Function

Tongue-lip motor function was evaluated using oral diadochokinesis (ODK). A participant was instructed to produce each of the syllables /pa/, /ta/, and /ka/ repeatedly for 5 s. The number of syllables produced per second was determined using an automatic counter (Kenkokun Handy, Takei Scientific Instruments Co., Ltd., Niigata, Japan) [22].

#### 2.3.5. Tongue Pressure

Maximum tongue pressure was measured using a tongue pressure measuring instrument (JMS TPM-01, JMS Co., Ltd., Hiroshima, Japan). The average of the three measurements was considered the tongue pressure of the participant [23].

#### 2.3.6. Masticatory Function

Masticatory function was evaluated by glucose concentration. Participants were asked to chew 2 g of gummy jelly; subsequently, the amount of eluted glucose was measured using a masticatory ability testing system (Gluco Sensor GS-II, GC Corporation, Tokyo, Japan) [24].

#### 2.3.7. Swallowing Function

A self-administered questionnaire for swallowing screening (the 10-item Eating Assessment Tool [EAT-10]) was used to assess swallowing function [25].

### 2.4. Data Analysis

Participants were divided by age (<75 years and ≥75 years). As the acquired ORF data were not normally distributed, the Mann–Whitney *U* test was used to examine the differences in KAOF for each question in both groups. The changes in the KAOF were tested using the Wilcoxon signed-rank test. Statistical analyses were performed using SPSS software (version 27.0; IBM, Armonk, NY, USA). The critical value for rejecting the null hypothesis was*p* < 0.05.

## 3. Results

**Figure 2:**
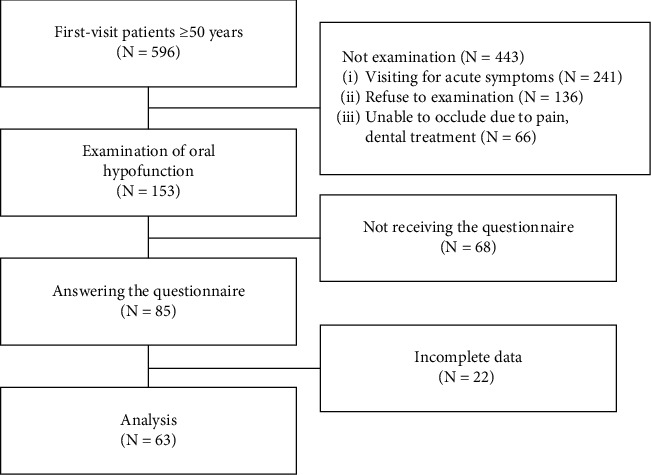
Schematic flow diagram of study participants.

A total of 596 patients were recruited for this study, and 63 patients (mean ± standard deviation [SD] age: 74.5 ± 11.1 years) were initially included (Figure 2).

The demographic data of the cohort are summarized in Table 3. Analysis 1 included 63 participants, of whom 28 were men and 16 performed care at home. Patients with a history of hypertension were the most numerous, followed by those with diabetes. Patients' educational background was most frequently high school. Of the 63 participants, four did not complete the questionnaire during the posttest. Therefore, Analysis 2 comparing before and after evaluation of ORF, included 59 participants, of whom 25 were men and 16 performed care at home. Hypertension and high school education were the most frequent.

### 3.1. Analysis 1: Relationship between KAOF and ORF before Evaluating ORF

The median (interquartile range) ORF values according to survey scores and age groups are presented in Table 4. For Question 1, all 26 participants aged < 75 years answered “do not know” or “do not understand.” Of the 37 participants aged ≥75 years, 33 responded negatively. For Question 2, EAT-10 was significantly higher (*p* = 0.036) in respondents aged < 75 years with positive answers. For Question 3, the TCI was significantly higher (*p* = 0.018) in participants aged < 75 years who responded negatively. Among those aged ≥75 years, ODK/pa/, /ta/, and /ka/ were significantly lower in patients who responded negatively (*p* = 0.002, 0.008, and 0.001, respectively); masticatory function was significantly higher in this group (*p* = 0.049). For Question 4, ODK/pa/ in the group aged < 75 years and ODK/ka/ among the participants aged ≥75 years were lower among participants who answered negatively (*p* = 0.047 and 0.033, respectively). For Question 5, in the ≥75-year group, oral dryness and occlusal force were significantly poorer in respondents who answered negatively (*p* = 0.022 and 0.025, respectively).

### 3.2. Analysis 2: Changes in KAOF

The distribution of responses before and after ORF evaluation is shown in Table 5. For all questions, the frequency of positive answers increased significantly after the evaluation (*p* < 0.001). For Question 1, on knowledge regarding oral hypofunction, positive answers comprised 6.8% in the initial dental interview and increased to 86.4% after ORF evaluation (*p* < 0.001), showing that the participants' knowledge of oral hypofunction improved. For Questions 2 and 3, the percentages of negative answers were 61.0% and 44.1%, respectively, before ORF evaluation and decreased to 25.4% and 22.0%, respectively, after evaluation (both *p* < 0.001). Through oral management, including evaluation, participants were able to understand their own ORF and recognize the need for training; furthermore, there was increased recognition that ORF is related to general health. For the questions about the need for evaluation of ORF (Questions 4 and 5), negative answers significantly decreased from 42.4% to 11.9% and from 35.6% to 11.9%, respectively, after oral management (both *p* < 0.001). The number of participants who wanted to have their ORF (in addition to caries and periodontal diseases) examined at the dental office increased.

## 4. Discussion

This study aimed to elucidate the relationship between KAOF and ORF in middle-aged and older individuals and the effect of management and motivation by dental practitioners on KAOF. The results indicated that older adults with poor KAOF had poor ORF. Furthermore, KAOF improved significantly after ORF evaluation and motivation. These results affirm the importance of KAOF, similar to previous reports that patients with poor dental knowledge had poor periodontal health [17, 18]. Patients with a poor understanding of oral health had poor oral hygiene and were found to improve their understanding of oral health and brushing frequency after education by dental hygienists [26]. According to the aforementioned studies, it is expected that ORF education will inculcate an understanding of ORF, resulting in improved ORF. For community-dwelling older adults, a decline in systemic and oral function is a serious health problem requiring long-term care [27]. Appropriate management of oral hypofunction is crucial for preventing these problems, as well as physical frailty and sarcopenia [28].

### 4.1. Relationship between KAOF and ORF before Evaluating ORF

For Question 1, which assessed understanding of oral hypofunction, 93.7% of the participants responded negatively, suggesting that public awareness of oral hypofunction was insufficient. Recently, in Japan, information about the relationship between general health and oral health has spread through various media, including the Internet and books. However, according to a previous survey in a Japanese region, only 11.6% of dental clinics in the area performed ORF examinations routinely [29]. Even in the survey by the training institutions of JSG, only 45 institutions (54%) measured ORF [30]. Therefore, awareness of oral hypofunction was low not only among general middle-aged and older adults but also among dental practitioners.

Individuals who responded positively to Question 2 tended to have poorer ORF than individuals who responded negatively. In the ≥75 year age group, individuals who responded positively showed poor ORF in four criteria: oral hygiene, oral dryness, tongue pressure, and swallowing function. Question 2 inquired about the necessity of training in ORF. Therefore, many participants who responded positively may be aware of their poor ORF and the need for training.

Individuals with negative responses to Question 3, on the relationship between ORF and general health, also showed poor tongue-lip motor function, especially those individuals ≥75 years old. Patients with positive KAOF may have higher health literacy and normally take care of their own health, including oral health. Since they are likely to be actively exercising for their oral and general health, regular feedback on their health status and suggestions for more effective exercises may be required. As it is evident that participants who responded negatively may not be aware of their poor ORF, it is important to provide health education.

For Questions 4 and 5, on the necessity of examining ORF, the ≥75 year group had a similar number of positive and negative responses; however, patients aged < 75 years had more positive responses, suggesting that the younger the age, the more healthconscious the participants were. Television programs and magazines include content on awareness regarding preventive medicine, such as the promotion of healthy lifestyles and the introduction and practice of simple exercises. In addition, preventive health education, including for ORF, should be provided to older individuals by professionals when they visit the dental office. This may improve healthy life expectancy. Our results also suggest that education on preventive healthcare is particularly important for those aged 75 years and above.

### 4.2. Changes in KAOF

The responses to Questions 1 to 5 significantly improved in terms of the ratio of positive responses after ORF evaluation. Therefore, the evaluation of ORF and awareness regarding oral hypofunction could have significantly influenced KAOF. As simple interventions, such as evaluation and education of ORF, can improve KAOF, it is important to motivate patients during dental office visits.

Some oral diseases have no noticeable symptoms and may progress gradually. If years pass without appropriate treatment, this may result in tooth loss. A decrease in the number of teeth has a significant effect on the decline in ORF, such as masticatory function and occlusal force. However, there are reports that masticatory function improves when occlusion is enhanced [31]. Tooth loss was associated with a lower intake of meat, fruits, and vegetables [32, 33], and a decline in occlusal force was associated with a lower intake of dietary fiber, most vitamins, and minerals [34]. Other reports suggest a relationship between poor ORF and malnutrition [35, 36]. Malnutrition, including a lower intake of proteins and vitamins, may worsen general health and decrease motor function and immunity, increasing the risk of musculoskeletal disorders and infections.

Temporomandibular joint disease can also make maintenance of oral hygiene and eating difficult. Fatigue and pain in opening and closing the mouth can lead to avoidance of hard foods and reluctance to eat [37]. In addition to affecting ORF, temporomandibular joint disease also decreases oral health satisfaction [38]. In this study, occlusal force and masticatory function were examined, but the status of the temporomandibular joint was not evaluated. Because there are systemic diseases that may affect the temporomandibular joint [39], it is important to carefully monitor medical histories when evaluating ORF in older adults and patients with systemic diseases. Since temporomandibular joint disease makes opening and closing the mouth, occlusion, and mastication difficult [37], it is recommended that temporomandibular joint symptoms also be identified when evaluating ORF.

The present study suggests that middle-aged and older adults with poor KAOF had significantly poorer ORF, especially ODK, which worsened with age. Although ORF declines with age, improving KAOF may help to slow the decline. In dental clinics, ORF assessment is recommended for middle-aged and older adults who show signs of declining ORF and who may be motivated by education that focuses on the impact of poor ORF and the need to maintain and improve it. In addition, patients and dental practitioners need to understand the importance of early evaluation and education. Oral health education will help end the cycle of frailty, slow down the speed of the cycle, and thus increase the healthy life expectancy of older adults.

### 4.3. Limitations

This study has several limitations. First, only short-term changes in KAOF, at the time of evaluation of ORF during a dental visit, were demonstrated. Participants were not necessarily patients who visited the dental office with ORF-related chief complaints. Therefore, routine management and follow-up of the participants were difficult. To maintain a good ORF, it is essential to maintain a positive KAOF through regular evaluation and education, and daily practice and training are required to maintain and improve ORF. Although KAOF can be improved by brief interventions, long-term and continuous management is desirable for sustained improvement, especially in patients with negative KAOF. Further studies with extended follow-up periods are warranted to elucidate the effect of ORF management on sustained positive KAOF.

Second, this study was observational and lacked a control group. The ORF was evaluated, followed by education, and then a postassessment was conducted. If we had included a control group of individuals who were not provided education regarding ORF, the KAOF of that group may or may not have improved. Therefore, it would have provided better insight into whether education regarding ORF in conjunction with ORF examination is better than ORF examination alone. Further studies are warranted to elucidate how ORF evaluation and education contribute to the improvement of KAOF.

## 5. Conclusion

Middle-aged and elderly individuals with poor KAOF were more likely to have worsening ORF, although KAOF could be improved by ORF assessment and education. These results indicate that to prevent ORF decline and frailty, it is important to evaluate the general condition of the individual, including medical history and nutrition status, as well as the oral health status, including occlusion and temporomandibular joint condition, from an early age, and that appropriate oral management and motivation are important. Furthermore, this study suggests that the general awareness of oral hypofunction may be low; therefore, more public awareness programs are needed.

## Figures and Tables

**Table 1:** Questionnaire on knowledge and attitude towards oral function (translated from Japanese).

Q1. I am familiar with the phrase “oral hypofunction.”
Q2. I believe that oral exercises are necessary for me.
Q3. Decreased oral function causes poor general health.
Q4. In addition to dental caries and periodontal disease screenings, I should get an oral function examination at the dental clinic.
Q5. I think it is important to evaluate oral function.

**Table 2:** Oral hypofunction criteria.

Oral function	Cut-off criterion
Oral hygiene	Tongue coating index ≥ 50%
Oral dryness	Measured value with a moisture checker < 27.0
Occlusal force	Occlusal force <500 N
Tongue-lip motor function	Utterance count of /pa/, /ta/, /ka/<6/s
Tongue pressure	Maximum tongue pressure <30 kPa.
Masticatory function	Glucose concentration in the chewing test <100 mg/dL
Swallowing function	Total score in 10-item eating assessment tool ≥ 3

**Table 3:** Demographic data of the participants.

Characteristic	Analysis 1 (*N* = 63)		Analysis 2 (*N* = 59)	
Mean ± SD age, years	74.5 ± 11.1		74.2 ± 10.9	
	<75 years	≥75 years	<75 years	≥75 years
*N*	26	37	25	34
Male/female	13/13	15/22	13/12	12/22
Outpatient/home care	25/1	22/15	24/1	19/15
Education, *N* (%)				
Junior high school	4 (15.4)	14 (37.8)	4 (16.0)	13 (38.2)
High school	11 (42.3)	15 (40.5)	10 (40.0)	15 (44.1)
Junior college	4 (15.4)	3 (8.1)	4 (16.0)	3 (8.8)
University	6 (23.1)	3 (8.1)	6 (24.0)	2 (5.7)
Graduate school	1 (3.8)	1 (2.7)	1 (4.0)	1 (2.9)
Not responded	0 (0.0)	1 (2.7)	0 (0.0)	0 (0.0)
Medical history, *N* (%)^†^				
Hypertension	3 (11.5)	16 (43.2)	3 (12.0)	15 (44.1)
Diabetes mellitus	2 (7.7)	6 (16.2)	2 (8.0)	6 (17.6)
Stroke	0 (0.0)	4 (10.8)	0 (0.0)	4 (11.8)
Cardiovascular diseases	2 (7.7)	5 (13.5)	2 (8.0)	5 (14.7)
Cancer	2 (7.7)	5 (13.5)	2 (8.0)	5 (14.7)
Others	10 (38.5)	15 (40.5)	9 (36.0)	15 (44.1)
Unknown	9 (34.6)	9 (24.3)	9 (36.0)	7 (20.6)

†Includes duplicated data.

**Table 4:** Medians (interquartile ranges) of parameters of oral function according to each question.

	*N* (%) of answers		Oral hygiene (TCI†; (%))	*p*value	Oral dryness	*p*value	Occlusal force (*N*)	*p*value	Tongue-lip motor function (*n*/*s*)						Tongue pressure (kPa)	*p*value	Masticatory function (mg/dL)	*p*-value	Swallowing function (EAT-10§)	*p*-value
									ODK‡/pa/	*p*value	ODK‡/ta/	*p*value	ODK‡/ka/	*p*value						
*Q1. I am familiar with the phrase “oral hypofunction.”*														
<75 years																				
Yes	0	(0.0)	—	N/A	—	N/A	—	N/A	—	N/A	—	N/A	—	N/A	—	N/A	—	N/A	—	N/A
No	26	(44.1)	28.0 (22.0–39.0)		27.9 (25.5–28.6)		445.8 (269.8–782.5)		6.6 (6.2–6.9)		6.6 (6.4–7.0)		6.1 (5.8–6.5)		36.7 (29.2–40.8)		172.0 (131.8–247.3)		0.0 (0.0–3.0)	
≥75 years																				
Yes	4	(6.8)	24.9 (13.8–28.0)	0.543	27.1 (26.4–28.0)	0.407	267.9 (161.9–267.9)	1.000	6.9 (5.5–7.2)	0.065	6.6 (5.2–7.3)	0.127	6.2 (5.6–6.7)	0.033^*∗*^	27.1 (23.5–30.8)	0.620	105.5 (92.8–222.5)	0.366	0.5 (0.0–1.8)	0.759
No	33	(55.9)	27.9 (12.3–27.5)		28.2 (24.7–29.3)		374.2 (204.6–718.9)		6.0 (5.2–6.2)		5.8 (5.2–6.0)		5.2 (4.8–5.8)		31.0 (19.8–34.3)		148.5 (115.3–196.3)		0.0 (0.0–2.0)	

*Q2. I believe that oral exercises are necessary for me.*														
<75 years																				
Yes	11	(18.6)	28.0 (22.0–39.0)	0.330	28.1 (27.6–28.7)	0.330	415.0 (206.4–646.5)	0.166	6.6 (6.0–7.4)	0.959	6.6 (6.4–6.8)	0.919	6.0 (5.8–6.4)	0.574	37.5 (29.2–38.3)	0.799	212.0 (140.0–268.0)	0.443	3.0 (0.0–6.0)	0.036^*∗*^
No	15	(25.4)	33.0 (22.0–39.0)		27.6 (25.2–28.6)		676.6 (381.3–944.2)		6.6 (6.4–6.8)		6.6 (6.2–7.0)		6.2 (5.6–6.6)		36.6 (29.1–51.5)		139.0 (131.0–245.0)		0.0 (0.0–1.0)	
≥75 years																				
Yes	13	(22.0)	28.0 (11.0–39.0)	0.948	27.0 (23.4–28.2)	0.132	425.1 (220.5–764.8)	0.595	6.0 (4.2–6.4)	0.937	5.8 (3.9–6.5)	1.000	5.3 (4.8–6.4)	0.728	29.9 (22.0–34.9)	0.695	181.0 (118.3–235.8)	0.128	2.0 (0.0–3.0)	0.212
No	24	(40.7)	27.8 (15.8–33.0)		28.4 (26.8–29.4)		359.3 (183.1–727.1)		6.0 (5.4–6.2)		5.9 (5.4–6.0)		5.3 (4.9–5.8)		30.9 (18.4–33.9)		135.5 (108.5–173.8)		0.0 (0.0–2.0)	

*Q3. Decreased oral function causes poor general health.*														
<75 years																				
Yes	15	(25.4)	22.0 (11.0–33.0)	0.018^*∗*^	27.8 (25.4–28.7)	0.799	415.0 (206.4–776.6)	0.397	6.6 (6.2–6.8)	0.919	6.6 (6.2–6.8)	0.646	6.0 (5.6–6.2)	0.259	36.7 (29.1–40.3)	1.000	147.0 (124.0–245.0)	0.413	0.0 (0.0–3.0)	0.721
No	11	(18.6)	39.0 (28.0–39.0)		28.1 (25.5–28.3)		603.0 (366.2–952.2)		6.8 (6.0–7.0)		6.6 (6.4–7.0)		6.4 (6.0–6.6)		36.6 (30.3–42.1)		225.0 (132.0–276.0)		0.0 (0.0–3.0)	
≥75 years																				
Yes	18	(30.5)	22.0 (11.0–28.0)	0.121	27.6 (26.0–28.8)	0.916	519.4 (183.3–794.1)	0.414	6.2 (5.9–6.8)	0.002^*∗*^	6.1 (5.7–6.7)	0.008^*∗*^	6.0 (5.1–6.4)	0.001^*∗*^	30.4 (23.3–33.4)	0.753	116.0 (103.0–177.5)	0.049^*∗*^	1.0 (0.0–2.3)	0.425
No	19	(32.2)	28.0 (16.7–50.0)		28.2 (24.3–29.6)		330.8 (219.8–423.3)		5.6 (4.4–6.0)		5.4 (5.0–6.0)		5.0 (3.4–5.4)		30.8 (17.8–34.2)		164.0 (134.0–198.0)		0.0 (0.0–2.0)	

*Q4. In addition to dental caries and periodontal disease screenings, I should get an oral function examination at the dental clinic*.																				
<75 years																				
Yes	18	(30.5)	28.0 (19.3–39.0)	0.144	28.0 (26.2–28.6)	0.531	464.2 (356.1–817.5)	0.534	6.8 (6.2–7.1)	0.047^*∗*^	6.7 (6.4–7.2)	0.080	6.1 (6.0–6.6)	0.495	37.0 (29.2–42.6)	0.461	205.0 (137.0–257.5)	0.285	0.0 (0.0–3.0)	0.683
No	8	(13.6)	36.0 (23.5–42.8)		27.8 (22.3–28.5)		445.8 (163.4–788.4)		6.5 (5.8–6.6)		6.5 (5.7–6.8)		6.1 (5.4–6.4)		34.2 (23.0–39.5)		135.5 (126.0–236.8)		0.0 (0.0–1.8)	
≥75 years		(0.0)																		
Yes	17	(28.8)	28.0 (16.3–36.0)	0.661	28.2 (27.3–28.9)	0.407	519.4 (253.3–811.5)	0.064	6.2 (5.3–6.4)	0.232	6.0 (5.3–6.3)	0.390	5.6 (5.3–6.4)	0.033^*∗*^	31.6 (27.1–35.8)	0.069	143.5 (113.8–199.3)	0.789	1.0 (0.0–2.0)	0.460
No	20	(33.9)	22.2 (11.0–33.0)		27.3 (24.3–29.6)		267.2 (169.2–381.6)		5.7 (5.1–6.2)		5.6 (5.2–6.2)		5.0 (4.7–5.8)		25.7 (16.8–32.1)		140.5 (106.8–192.0)		0.0 (0.0–2.0)	

*Q5. I think it is important to evaluate oral function*.																				
<75 years																				
Yes	21	(35.6)	28.0 (22.0–39.0)	0.753	27.7 (25.5–28.5)	0.447	504.2 (269.8–864.4)	0.496	6.6 (6.2–6.9)	0.801	6.6 (6.3–7.1)	0.705	6.0 (5.7–6.5)	1.000	36.6 (29.1–41.1)	0.801	225.0 (128.5–261.0)	0.308	0.0 (0.0–3.0)	0.486
No	5	(8.5)	33.0 (16.5–47.3)		28.2 (24.6–29.8)		410.7 (241.4–630.1)		6.6 (5.9–7.2)		6.6 (6.0–6.9)		6.2 (5.7–6.4)		37.7 (25.4–47.0)		138.0 (131.5–198.5)		0.0 (0.0–3.0)	
≥75 years																				
Yes	19	(32.2)	28.0 (15.8–39.0)	0.661	28.4 (27.6–29.6)	0.022^*∗*^	519.4 (316.5–811.5)	0.025^*∗*^	6.0 (4.4–6.2)	0.620	5.8 (5.2–6.0)	0.599	5.3 (4.8–5.9)	0.767	31.6 (23.6–34.6)	0.178	163.0 (115.8–199.0)	0.323	1.0 (0.0–2.0)	0.271
No	18	(30.5)	22.2 (11.1–33.0)		26.4 (24.3–28.4)		235.6 (172.5–336.5)		6.0 (5.4–6.7)		5.9 (5.4–6.3)		5.4 (4.8–6.1)		26.5 (17.2–32.4)		131.0 (100.3–180.3)		0.0 (0.0–2.0)	

†TCI: tongue coating index, ‡ODK: oral diadochokinesis, §EAT-10: the 10-item eating assessment tool. ^*∗*^*p* < 0.05.

**Table 5:** Changes in responses to each question.

	Before examination and education *N* (%)	After examination and education *N* (%)	*p* value
Q1. I am familiar with the phrase “oral hypofunction.”			
Understand well	0 (0)	23 (39.0)	<0.001^*∗*^
Know	4 (6.8)	28 (47.5)	
Do not know	8 (13.6)	5 (8.5)	
Do not understand	47 (79.7)	3 (5.1)	

Q2. I believe that oral exercises are necessary for me.			
Strongly agree	5 (8.5)	27 (45.8)	<0.001^*∗*^
Agree	18 (30.5)	17 (28.8)	
Disagree	9 (15.3)	11 (18.6)	
Strongly disagree	27 (45.8)	4 (6.8)	

Q3. Decreased oral function causes poor general health.			
Strongly agree	12 (20.3)	21 (35.6)	<0.001^*∗*^
Agree	21 (35.6)	25 (42.4)	
Disagree	8 (13.6)	10 (16.9)	
Strongly disagree	18 (30.5)	3 (5.1)	

Q4. In addition to dental caries and periodontal disease screenings, I should get an oral function examination at the dental clinic.			
Strongly agree	8 (13.6)	27 (45.8)	<0.001^*∗*^
Agree	26 (44.1)	25 (42.4)	
Disagree	12 (20.3)	5 (8.5)	
Strongly disagree	13 (22.0)	2 (3.4)	

Q5. I think it is important to evaluate oral function.			
Strongly agree	13 (22.0)	33 (55.9)	<0.001^*∗*^
Agree	25 (42.4)	19 (32.2)	
Disagree	11 (18.6)	6 (10.2)	
Strongly disagree	10 (16.9)	1 (1.7)	

^*∗*^ *p* < 0.05.

## Data Availability

The data that support the findings of this study are available from the corresponding author upon reasonable request.
